# Deep Learning-Powered Down Syndrome Detection Using Facial Images

**DOI:** 10.3390/life15091361

**Published:** 2025-08-27

**Authors:** Mujeeb Ahmed Shaikh, Hazim Saleh Al-Rawashdeh, Abdul Rahaman Wahab Sait

**Affiliations:** 1 Department of Basic Medical Science, College of Medicine, AlMaarefa University, Diriyah 13713, Riyadh, Saudi Arabia; 2 King Salman Center for Disability Research, Riyadh 11614, Saudi Arabia; hazim@oc.edu.sa; 3 Cyber Security Department, College of Engineering and Information Technology, Onaizah Colleges, Onaizah 56447, Qassim, Saudi Arabia; 4 Department of Archives and Communication, Center of Documentation and Administrative Communication, King Faisal University, P.O. Box 400, Hofuf 31982, Al-Ahsa, Saudi Arabia

**Keywords:** facial images, feature fusion, explainable down syndrome detection, deep learning, SHAP, chromosomal abnormalities

## Abstract

Down syndrome (DS) is one of the prevalent chromosomal disorders, representing distinctive craniofacial features and a range of developmental and medical challenges. Due to the lack of clinical expertise and high infrastructure costs, access to genetic testing is restricted to resource-constrained clinical settings. There is a demand for developing a non-invasive and equitable DS screening tool, facilitating DS diagnosis for a wide range of populations. In this study, we develop and validate a robust, interpretable deep learning model for the early detection of DS using facial images of infants. A hybrid feature extraction architecture combining RegNet X–MobileNet V3 and vision transformer (ViT)-Linformer is developed for effective feature representation. We use an adaptive attention-based feature fusion to enhance the proposed model’s focus on diagnostically relevant facial regions. Bayesian optimization with hyperband (BOHB) fine-tuned extremely randomized trees (ExtraTrees) is employed to classify the features. To ensure the model’s generalizability, stratified five-fold cross-validation is performed. Compared to the recent DS classification approaches, the proposed model demonstrates outstanding performance, achieving an accuracy of 99.10%, precision of 98.80%, recall of 98.87%, F1-score of 98.83%, and specificity of 98.81%, on the unseen data. The findings underscore the strengths of the proposed model as a reliable screening tool to identify DS in the early stages using the facial images. This study paves the foundation to build equitable, scalable, and trustworthy digital solution for effective pediatric care across the globe.

## 1. Introduction

Down syndrome (DS), referred to as trisomy 21, is caused by the inheritance of an additional copy of chromosome 21 [1]. This genetic variation may impact Physiological and cognitive development, leading to various medical conditions, including endocrine irregularities, increased susceptibility to infections, and congenital heart defects [2]. Prenatal screening and invasive procedures, such as amniocentesis, are used to detect DS in the early stages [3]. These standard testing procedures have been shown to be effective in diagnosing chromosomal abnormalities [3]. However, these procedures have a certain degree of risk, expense, and logistical complexity. Thus, noninvasive approaches are gaining popularity in order to minimize patient discomfort and reduce resource-intensive steps [4]. Due to the significance of unique facial characteristics, artificial intelligence (AI)-driven diagnostic tools utilize facial phenotyping to identify DS [5].

Clinical observation and handcrafted features have provided a level of dependability [6,7,8]. However, the outcomes of these approaches are based on the clinical expertise of healthcare professionals. Early approaches require precisely managed settings, including image capturing at consistent angles, uniform lighting, and regulated backdrop settings, making them unsuitable for real-world applications [9]. Additionally, traditional feature extraction pipelines face challenges in diagnosing patients due to physical variability between age groups or ethnicities [10]. Conventional techniques involve laborious manual assessments, which increase the complexity of large-scale screening [11]. These techniques form the foundation of medical diagnostics, enabling us to comprehend phenotypic indicators. However, they fail to satisfy scalability, flexibility, and high-throughput accuracy requirements.

Early DS diagnosis is essential to initiate timely medical and educational interventions, improving the individual’s quality of life [12]. These prenatal screening modalities, which include noninvasive prenatal testing and invasive procedures, provide valuable opportunities for early risk assessment [12]. However, they are not universally accessible and may be declined or unavailable due to factors, such as cost, cultural factors, or limited resources. In certain populations, they may be declined or unavailable due to factors such as cost, cultural factors, or limited resources. Consequently, the majority of individuals with DS are diagnosed after birth, highlighting the continued clinical significance of accessible and effective postnatal diagnostic methods. Physical examinations by experienced clinicians and cytogenetic confirmation are reliable standard postnatal diagnostic pathways in healthcare settings with sufficient resources [13]. However, in underserved or remote areas, access to specialized expertise and timely laboratory facilities is limited, making these pathways less feasible. This emphasizes the necessity for non-invasive techniques to detect DS early in the postnatal period, especially in situations where diagnosis delays could delay intervention and family counseling.

According to existing studies [13,14,15], managing clinical difficulties at an earlier stage can lead to improved health outcomes and effective integration into broader social contexts. Although genetic or chromosomal testing is highly accurate, its application is restricted in resource-constrained clinical settings. The reliance on manual assessment may lead to missed or delayed diagnosis. The emergence of facial phenotyping supports syndromic recognition in neonates and infants, leading to the development of artificial intelligence-driven DS diagnosis [16]. Developing a robust DS screening system to detect subtle variations in facial image features is an intriguing frontier in computer vision and pattern recognition, motivating us to reevaluate DL architecture designs, incorporate novel data augmentation strategies, and investigate approaches to enable model interpretation [17]. The combination of these practical and technological motivators highlights the relevance of this research initiative, alleviating the responsibilities of families and healthcare professionals by providing a simplified and cost-effective DS screening model. Existing DS detection approaches have frequently emphasized classification accuracy over interpretability and generalizability [18]. There is a demand for an ideal combination of optimal classification performance and a generalizable model architecture for developing a DS detection model for diverse populations.

In recent years, Deep learning (DL) techniques have emerged as a promising tool for automating image analysis, minimizing noise, and enhancing diagnostic efficiency in biomedical fields. Yu et al. [19] revealed that deep learning models can rapidly denoise and filter ultrasound images, improving image quality and diagnostic potential in ultrasonic localization microscopy. Similarly, Cao et al. [20] demonstrate the significance of deep learning techniques in the healthcare domain by revealing intricate biological processes through the application of sophisticated computational methods. The recent DL techniques, including convolutional neural networks (CNN), autoencoders, and vision transformers (ViT), have gained popularity in medical imaging applications [21]. By applying these models to the DS identification, we can automatically extract multidimensional face features. CNN and ViT architectures have the potential to distinguish DS-specific features from typical developmental patterns [22]. However, these architectures encounter significant challenges. The lack of large-scale annotated DS datasets reduces the classifier’s generalization ability to a diverse population [23]. Furthermore, a considerable number of deep neural networks comprise millions of trainable parameters, increasing the risk of model overfitting. Computational training and inference overhead could lead to operational challenges in resource-limited healthcare settings. These challenges highlight the need to develop an innovative hybrid model that incorporates the complementary advantages of CNNs and ViTs architecture, maximizing classification performance without compromising interpretability.

Reliability, scalability, and less invasive techniques are crucial for enhancing the early and accurate diagnosis of DS utilizing face imaging [24]. The existing DL approaches have shown some effectiveness in extracting latent facial signals. However, their limitations include a lack of well-annotated datasets, model overfitting, and limited generalizability and interpretability. DS symptoms may manifest differently depending on age group, ethnic background, and imaging environment [25]. Existing models demand substantial data augmentation and domain-specific calibration to handle this variability. These models employ standalone or single-feature extraction procedures, neglecting global representation. In addition, without focusing on clinical interpretability and age-specific performance, the DL-based DS screening models may not foster clinicians’ trust.

To address the shortcomings of the existing approaches, an automated DS detection is developed using facial images. The proposed model leverages a unique combination of DL architectures, adaptive fusion, bias mitigation, and explainability strategies to address the shortcomings of the existing methodologies. Although there are standard genetic testing procedures accessible in healthcare systems, such as karyotyping or chromosomal microarray analysis, the timely and widespread application of these technologies is hindered by practical limitations. For instance, the requirement for intrusive sample collection (e.g., venipuncture), high per-test costs, lengthy laboratory turnaround times, and reliance on specialized infrastructure and trained professionals are significant challenges in traditional DS diagnostics based on karyotyping. In clinical settings, these challenges may delay early DS diagnosis. Through the proposed model, clinicians may streamline patient flow and resource allocation by prioritizing at-risk patients for confirmatory genetic testing. Additionally, the tool can be used as a first-line screening method in outreach programs, school-based screenings, or rural healthcare settings. The proposed model is intended to support routine, accurate, and minimally invasive postnatal diagnostic practices, including physical examination and karyotyping in settings where clinical expertise or immediate cytogenetic confirmation is limited. The innovative contributions of this study are outlined as follows:

1. Hybrid feature extraction using RegNet-X–MobileNet V3 and ViT-Linformer.

The proposed feature extraction integrates the powerful spatial inductive biases of convolution networks with the global contextual reasoning capabilities of ViT architectures. It enables the DS classification model to comprehensively learn local and holistic facial characteristics associated with DS phenotyping, presenting a robust and developmentally sensitive feature representation.

2. Adaptive attention-based feature fusion with cross-demographic bias mitigation layer.

The adaptive attention-based fusion dynamically prioritizes feature maps based on region saliency and diagnostic relevance. This module is used to reweight features extracted from RegNet-X–MobileNet V3 and ViT-Linformer. To address algorithm bias, we introduce a cross-demographic mitigation layer during the training phase. This layer minimizes performance disparity across ethnic, age, and gender subgroups. A demographic-aware loss function is employed to equalize the influence of classes during gradient updates.

3. Fusion-aware explainability enabled Extremely Randomized Trees (ExtraTrees) for DS classification.

The replacement of traditional dense output layers with an ExtraTrees classifier enables faster computation through the fully randomized node splits. The integration of fusion-aware explainability with Shapley additive explanations (SHAP) facilitates granular attribution of diagnostic relevance to specific regions and feature types, improving the clinical adoption of the proposed DS classification in healthcare.

The remaining sections of this study are organized into five sections, presenting the significance of the proposed DS detection framework. Section 2 critically examines the existing DS detection approaches using facial images. It highlights their limitations in generalization, interpretability, and fairness. Section 3 describes the proposed feature extraction, fusion, and classification methodology. The experimental results are outlined in Section 4. Section 5 provides an in-depth discussion of the experimental outcomes, model behavior, and interpretability insights. Finally, Section 6 concludes the study by summarizing key findings and their implications and outlining directions for future work in developing DS detection models.

## 2. Literature Review

Facial imaging is becoming an increasingly effective and non-invasive diagnostic tool [26]. As a result, a significant amount of research has been focused on automating DS identification using advanced AI techniques. The existing approaches range from traditional machine learning techniques with handcrafted features to recent advances utilizing CNN and ViT architectures [27]. Conventional approaches suffer from critical technical limitations, which reduce their effectiveness in clinical settings [28]. The classical image processing techniques, including geometric distances, texture descriptors, and shape-based metrics, typically extract pre-defined features from facial images [28]. These approaches are unable to extract novel or abstract facial patterns, limiting their ability to identify subtle DS phenotypic markers. Handcrafted features may represent low-dimensional and sparse representations of the facial image, restricting the expressiveness of the input to the classifier [28]. Moreover, traditional approaches are highly sensitive to variations in lighting and age-related facial morphology.

Mittal et al. [28] employ local binary patterns to extract facial features from two-dimensional images. They achieve moderate accuracy with limited robustness to real-world variation. Pooch et al. [29] propose a framework using facial landmarks and geometric measurements. However, the dataset size and diversity remain limited, restricting generalization across diverse populations. Qin et al. [30] enhance the pre-trained face recognition networks, achieving an accuracy of 95.87% with effective recall and specificity. The experimental outcomes indicate the CNN’s potential in capturing the facial morphology characteristics of DS. Wang et al. [31] use the ResNet64 model with a squeeze-and-excitation block for DS detection. They apply cross-loss training and robust pre-processing approaches.

Gerenek and Szklanny [32] evaluate the performance of CNNs, including ArcFace, DeepFace, FaceNet, and other pre-trained CNNs. There is a lack of information on geometric analysis and demographic bias. Porras et al. [33] and Islam and Shaikh [34] enhance pre-trained CNNs by incorporating an additional attention mechanism to extract subtle DS patterns from the facial images. Raza et al. [35] introduce a hybrid architecture using VGG-16 with non-negative matrix factorization. They report near-perfect accuracy of 99.00% on a dataset of 3009 facial images. Nevertheless, the model’s interpretability and fairness mechanisms were limited. The feature fusion occurred at a high level, neglecting nuanced interactions between different representation layers.

Despite promising findings, the recent approaches have significant shortcomings, including limited generalizability, lack of interpretability, and demographic bias. CNNs pose a considerable limitation in capturing long-range dependencies and global facial symmetry, which are crucial for DS diagnosis. The real-time facial images may cause challenges to CNNs due to a low resource environment and variations in image quality. The performance of CNNs may drop significantly under non-ideal conditions unless the dataset is large and highly diverse. Emphasizing dominant visual features can enable the CNNs to neglect crucial DS features. ViTs can capture long-range dependencies and global contextual information. The generalizability of existing models lacks representation of diverse age ranges and imaging conditions, resulting in model overfitting. The existing gaps can be addressed by integrating ViT’s architecture to complement CNNs’ features, leading to accurate and explainable classifications. The inclusion of ViTs in the DS detection pipeline is a novel step in building clinically relevant, fair, and high-performance DS detection models.

## 3. Materials and Methods

**Figure 1:**
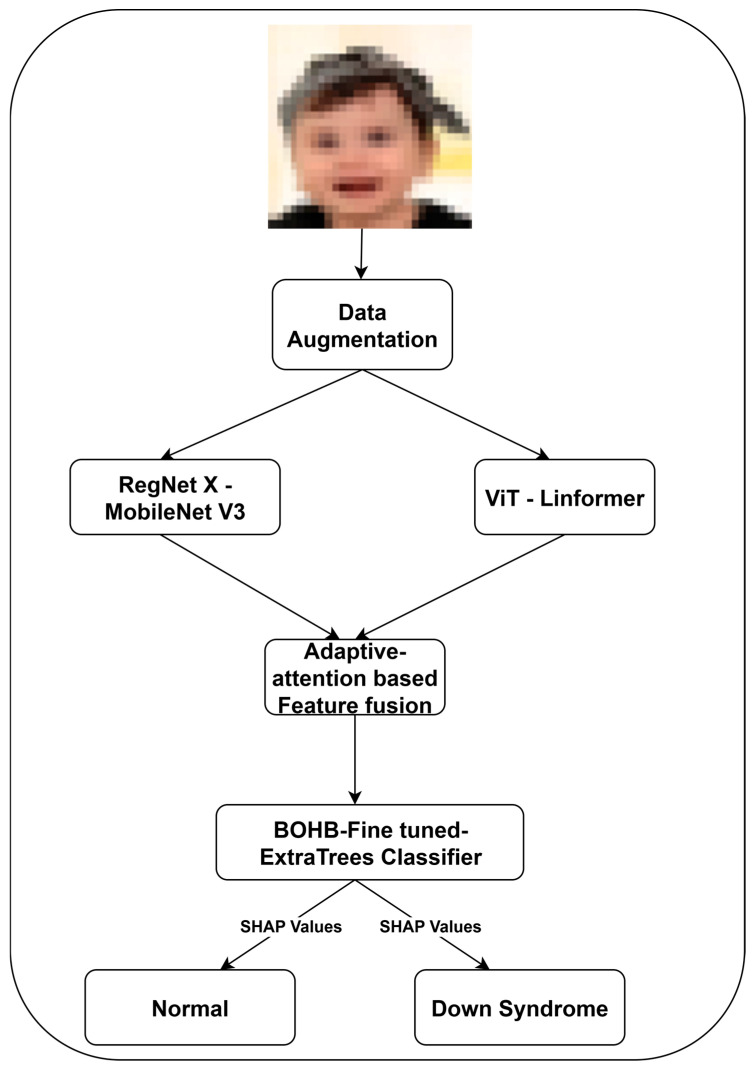
The Proposed Approach for DS Classification.

The craniofacial features associated with DS can vary significantly across individuals, causing challenges to DS classifiers. The combination of CNNs and ViTs integrates local features and global context understanding, motivating us to employ a hybrid RegNet X–MobileNet V3 and ViT-Linformer feature extraction strategy. By leveraging the complementary information, feature fusion enhances the robustness of the proposed model. Optimizing ExtraTrees through BOHB with SHAP can offer a reliable DS classification, facilitating the model’s generalizability and interpretability. Figure 1 illustrates the detailed methodology of DS classification using the facial images.

### 3.1. Data Acquisition and Pre-Processing

In this study, we utilize a publicly available dataset [36] that comprises 2999 facial images (1499 and 1500 individuals without and with DS) within the age range of 0 to 15 years. The dataset providers collected images from the hospital archives, obtained ethical clearance, and validated the datasets through healthcare professionals. The dataset includes ethnicities, such as Caucasian, East Asian, South Asian, Hispanic, and Indigenous or mixed-race, enabling the proposed model to incorporate a cross-demographic bias mitigation layer during the training phase. It spans various image qualities and environmental settings, including lighting, background clutter, and facial orientations, providing practical training for the hybrid feature extraction architectures and reducing the risks of overfitting. Pre-trained CNNs and ViTs can extract features from the raw images without extensive pre-processing. However, we apply preprocessing techniques, including normalization, alignment, and resizing, in order to maximize the performance of RegNet X [37]-MobileNet V3 [38] and ViT [39]-Linformer [40].

The dataset is retrospectively categorized into different age groups using metadata. This categorization allows us to analyze the infant subgroup, affirming the model’s applicability in early pediatric care. Table 1 highlights the details of the categorized dataset.

In face image-based phenotyping, age-related morphological changes such as facial elongation, skeletal growth, and soft tissue development may affect craniofacial markers. In addition, the broader age group may introduce non-DS heterogeneity may affect the performance of feature extraction pipelines. To reduce non-discriminative variance and improve the accuracy of the model’s learnt representations, we narrow the dataset’s age range from 0–15 years to the infant subgroup (0–2 years). Using age-consistent facial geometry, the feature extraction modules enable the attention-based feature fusion method to highlight actual syndrome-specific indicators without being distracted by age-induced noise.

Compared to the traditional train–test split, K-fold cross-validation supports the development of reliable healthcare applications, mitigating sampling bias and evaluating model stability across the population. Multiple studies have reported the significance of K-fold cross-validation in clinical artificial intelligence-powered models, providing better error estimation in class-imbalance datasets [41,42,43,44]. Thus, a stratified five-fold cross-validation is applied on the infant subgroup (0–2 years), reflecting the critical window for early phenotypic assessment. Each fold maintains a balanced distribution of DS and typical cases, addressing the risk of class imbalance.

Data augmentation plays a crucial role in improving the model’s ability to generalize and prevent overfitting. We employ innovative data augmentation techniques to increase the dataset size and diversity. With traditional methodologies, including random cropping, rotation, flipping, and scaling, we use sophisticated transformation methods, such as simulated midface flattening, slanting eyes, and other DS facial characteristics. These augmentation approaches support us in replicating the facial variations associated with individuals with DS. In addition, facial region-specific augmentations emphasizing the eyes, nose, and mouth areas are used to enhance the diversity of facial images. By learning a wider range of facial features, the proposed model can handle the subtle variations between individuals.

### 3.2. RegNet X—MobileNet V3-Based Feature Extraction

**Figure 2:**
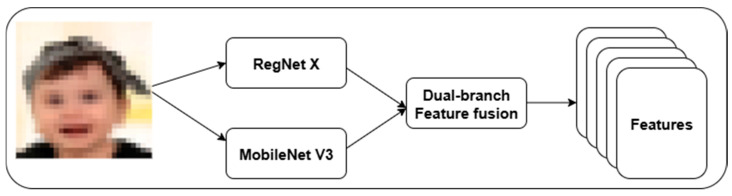
The Proposed RegNet X–MobileNet V3-based Feature Extraction.

We introduce a dual-branch feature extraction framework using RegNet X and MobileNet V3. Figure 2 highlights the proposed feature extraction approach. This strategy addresses the limitations of single-path convolutional models in existing DS classification. RegNet X provides a scalable high-level semantic representation, while MobileNet V3 offers low-level texture and edge feature extraction. In RegNet X architecture, a regularized design space for convolutional networks is used to control the number of channels, blocks, and stage-wise bottlenecks.

To reduce computation while balancing depth, RegNet X uses a bottleneck transformation with group convolutions. Equation (1) outlines the feature extraction process using RegNet X.

(1) $$F_{R}\left(x\right)=B\left(ReLu\left({Conv}_{1\times 1}\left(B\left(ReLu\left({Conv}_{3\times 3}\left(B\left(ReLu\left({Conv}_{1\times 1}\left(x\right)\right)\right)\right)\right)\right)\right)\right)\right)$$

where $F_{R}\left(x\right)$ is the feature, B is the bottleneck transformation, ReLu is the rectified linear unit, Conv is the convolutional layer, and x is the facial image.

MobileNet V3 is designed for mobile and edge devices. It integrates depthwise separable convolutions with squeeze and excitation (SE) modules and hard-swish activations, maximizing accuracy with minimal computational resources. Equation (2) is the computational form of MobileNet V3-based feature extraction.

(2) $$F_{M}\left(x\right)=AM\left(BN\left({Conv}_{1\times 1}\left(\sigma \left(BN\left({DWConv}_{3\times 3}\left(BN\left({Conv}_{1\times 1}\left(x\right)\right)\right)\right)\right)\right)\right)\right)$$

$F_{M}\left(x\right)$ is the MobileNet V3 feature, AM is the attention mechanism, BN is the inverted residual with a linear bottleneck, σ is the hard-swish activation function.

The proposed architecture focuses on fine-grained facial textures critical in distinguishing DS phenotypes from normal individuals. A dual-branch feature fusion is used to combine the high-capacity semantic abstraction of RegNet X with the lightweight MobileNet V3 models. Equation (3) represents the feature fusion process using a learned attention gate.

(3) $$F_{f}=\alpha ⨀F_{R}+\left(1+\propto \right)⨀F_{M}$$

where ⨀ denotes element-wise multiplication, $\alpha =\;\sigma \left({Conv}_{1\times 1}\left(\left[{\;F}_{R}∥{\;F}_{M}\right]\right)\right)$ is the adaptive attention mask learned from concatenated features.

The suggested adaptive fusion gate allows the model to focus on structural or textural features by balancing the contribution of each stream based on the image content. It aligns better with the visual complexity of DS presentation, addressing intra-class variance effectively compared to base models, including RegNet X and MobileNet V3.

### 3.3. ViT-Linformer-Based Feature Extraction

RegNet X–MobileNet V3 effectively extracts localized DS features. However, they struggle to capture long-range spatial dependencies and global contextual relationships between regions that are essential in facial dysmorphology analysis for DS detection. In order to address the limitations of RegNet X–MobileNet V3 models, an efficient attention approximation mechanism is developed using a hybrid ViT-Linformer model. The proposed ViT-Linformer processes the image as a unified entity, detecting DS distributed and relational features. The ViT component learns long-range dependencies and spatial relationships among facial features. These features are used to identify complex syndromic traits associated with DS. Images are divided into a sequence of non-overlapping patches. Each patch is flattened and projected into a latent embedding space using a linear transformation. A multiple transformer encoder layer processes the sequence using a multi-head self-attention mechanism. However, this self-attention mechanism has a quadratic time and space complexity, which may pose a computational bottleneck for high-resolution images. To address this shortcoming, we integrate the Linformer model that approximates self-attention using low-rank projections. In addition, the Linformer model uses learnable projection matrices to reduce dimensionality. The classification tokens $\left(Z_{CLS}^{\left(L\right)}\right)$ are extracted as the final feature representation by multiple attention and feed-forward layers. Equation (4) represents the feature extraction through ViT-Linformer.

(4) $$F_{ViT}=Z_{CLS}^{\left(L\right)}$$

where $F_{ViT}$ is the final feature representation.

The extracted feature vector covers a high-level, holistic representation of a face, encoding spatial dependencies across the entire image in a single embedding.

### 3.4. Feature Fusion

We design the feature fusion approach in order to guarantee semantic consistency, dimensional alignment, and fairness across demographic groups. A fully connected linear layer is used to perform dimensionality reduction and alignment. It projects the features of RegNet X- MobileNet V3 $\left(F_{f}\right)$ and ViT-Linformer ${(F}_{ViT})$ into a common latent space. The aligned feature vectors are processed through an adaptive attention gate, assigning dimension-wise importance to each modality. Equation (5) shows the fusion process that combines features, including $F_{f}$ and $F_{ViT}$.

(5) $$F_{F}=\alpha ⨀\hat{F_{f}}+\left(1+\propto \right)⨀\hat{F_{ViT}}$$

where $F_{F}$ is the final set of fused features, α is the adaptive attention gate, ⨀ is the element-wise multiplication, $\hat{F_{f}}$ and $\hat{F_{ViT}}$ are the features of RegNet X–MobileNet V3 and ViT-Linformer, respectively.

To guarantee fair and unbiased classification across different subpopulations, the final feature vector is passed through a cross-demographic bias mitigation layer during training. Equation (6) presents the computation of the group-balanced demographic-aware loss.

(6) $$L_{fair}=\sum_{g\leftarrow G}{W_{g}\cdot Ε}_{\left(x,y\right)∽D_{g}}\left[-ylog\hat{y}-\left(1-y\right)log\left(1-\hat{y}\right)\right]$$

where $L_{fair}$ is the total fairness-aware loss function, g is a demographic group and G is the set of groups, “·” is the multiplication operator, $W_{g\;}$ is the weight assigned to g, $D_{g}$ is the subset of the dataset containing only samples from g, $\left(x,y\right)∽\;D_{g}$ is an input-label pair from group g′s data, and $\hat{y}$ is the predicted probability output by the model.

### 3.5. DS Classification Using Fine-Tuned ExtraTrees Classifier

In this study, we employ ExtraTrees to introduce randomness in feature selection and split thresholding to reduce variance and mitigate overfitting. Equation (7) shows the selection process of the best threshold.

(7) t*=Ginixj<ttargmin

where $t^{*}$ is the best threshold from a set $\left\{t_{1},t_{2,}\ldots .,t_{k}\right\}$, and $x_{j}$ is a feature.

To optimize the ExtraTrees performance and reduce manual trial and error in hyperparameter tuning, BOHB is integrated with it. The ExtraTrees hyperparameters, including n-estimators, max-depth, min-samples-split, and max-features, are explored by BOHB using Bayesian surrogate modeling with early-stopping via successive halving. This approach maintains a balance between exploration and efficiency, which is valuable in clinical models requiring generalizability across diverse populations.

To ensure transparency in clinical decision-making, SHAP values are incorporated with the classifier, an importance score $\phi_{j}$ is assigned to each feature *j* in the fused vector, quantifying the individual contribution to the model’s decision. Equation (8) presents the computational form of the importance score for features.

(8) $$\phi_{j}=\sum_{S\subseteq F\backslash \left\{j\right\}}\frac{\left|S\right|!\left(\left|F\right|-\left|S\right|-1\right)!}{\left|F\right|!}\left[f\left(S⋃\left\{j\right\}\right)-f\left(S\right)\right]$$

where F is the complex set of features, $\;S\;\subseteq F\backslash \left\{j\right\}$ is a subset of the set of all features excluding feature *j*, and $f\left(S\right)$ is the model’s decision using the feature in S.

We apply the SHAP values to the fused representation, enabling global interpretation and local explanation. This dual-level explainability improves model accountability and supports clinical validation and trustworthiness.

### 3.6. Experimental Environment

The experiments are conducted on a system configured with Windows 11 Pro, Python 3.8.10, and NVIDIA GPU with CUDA 11.3, supported by 32 GB RAM. The proposed model architecture is implemented in PyTorch 2.4, utilizing the timm library for transformers backbones. Table 1 reveals the detailed implementation setup. The training strategy involved five-fold cross-validation with each fold preserving class balance and demographic distribution across five ethnic groups. During the training phase, extensive data augmentation was applied on the first four folds in order to simulate real-world variance and improve robustness. The fifth fold is used for generalization using raw and unaugmented samples, validating model under deployment-like conditions.

A comprehensive set of performance metrics are used to evaluate the model, guaranteeing diagnostic accuracy and clinical reliability. The exceptional accuracy, precision, recall, and F1-score indicate a well-balanced ability to detect DS and normal individuals with minimal false positives or negatives. The specificity score confirms the model’s capability to correctly identify normal individuals. The area under precision-recall curve (AUPRC) reflect the model’s discrimination capacity under class imbalance. The standard deviation and confidence intervals demonstrate model stability and consistency across diverse subsets of data. Additionally, the computational efficiency is revealed through the number of parameters and floating-point operations (FLOPs). Table 2 offers the configurations for implementing the proposed model.

## 4. Results

Table 3 outlines the best hyperparameters of the ExtraTrees classifier to achieve optimal performance in accuracy and fairness. Due to their direct and substantial effects on the model’s complexity, ability to capture non-linear relationships, and tendency to overfit or underfit the data, a subset of high-impact hyperparameters, including the number of estimators, maximum depth, minimum samples split, minimum samples leaf, learning rate, and regularization strength, are optimized through the BOHB optimization. We optimized the model design to enhance classification accuracy while decreasing bias and variance, resulting in reliable and interpretable results. To guarantee repeatability of outcomes, a fixed random state value was used in model training and evaluation experiments. During the processes of data splitting, model initialization, and resampling, the random state is responsible for determining the initialization of random number generators. The choice of 300 estimators offers a robust trade-off between prediction reliability and computational cost. A maximum depth of 20 allows the tree to capture complex, non-linear decision boundaries without overfitting. The values of min-samples-split and min-samples-leaf are essential for regulating the branching structure of the trees, preventing data sparsity from resulting in overfit leaf nodes. These hyperparameters contribute to achieving high classification performance, enabling SHAP-based explanation through tree-based inference.

Table 4 presents the outcomes of the five-fold cross-validation, highlighting the robustness, consistency, and generalization capability of the proposed model across five folds. The outcomes of the fifth fold reflect the model’s ability to perform well in a real-time DS screening environment that lacks controlled augmentation or preprocessing. The use of diverse features enhances the model’s discriminative power in identifying normal individuals, reducing unnecessary anxiety or referrals. The proposed model achieves consistent and transferable outcomes, reinforcing its generalization strength in facial-based syndromic analysis. The experimental findings set the model as a valuable non-invasive screening tool, enabling timely intervention and improved patient outcomes.

**Figure 3:**
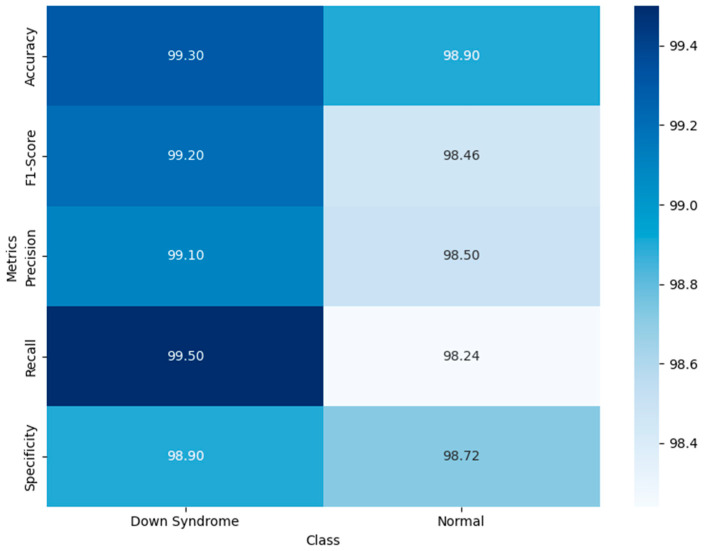
Findings of Performance Evaluation Using Heatmap by Class.

Figure 3 visualizes the model’s balanced and robust binary classification performance. The high recall value for DS cases guarantee the model’s ability to minimize false positives, minimizing unnecessary follow-up. It demonstrates the model’s balanced predictive ability, reflecting a reliable diagnostic ability across diverse populations. The high recall for DS guarantees that affected individuals are not overlooked. The attention-based hybrid architecture enhances the model’s classification ability, demonstrating its trustworthiness and suitability for population-wide screening.

**Figure 4:**
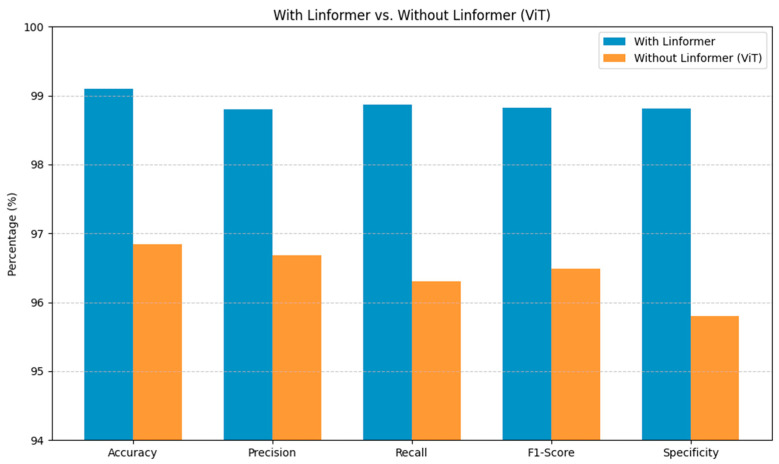
Performance of the Proposed Model with Linformer and without Linformer.

Figure 4 highlights the contextual value of incorporating the Linformer architecture within the proposed DS screening model. The Linformer-powered model consistently surpasses the standard ViT across the primary evaluation metrics. These substantial gains in the model’s performance are crucial for reliable identification of DS, minimizing diagnostic uncertainty. Integrating the Linformer’s attention mechanism addresses the shortcomings of the traditional ViT architectures, enabling efficient processing of high-resolution facial images in capturing the long-range dependencies.

**Figure 5:**
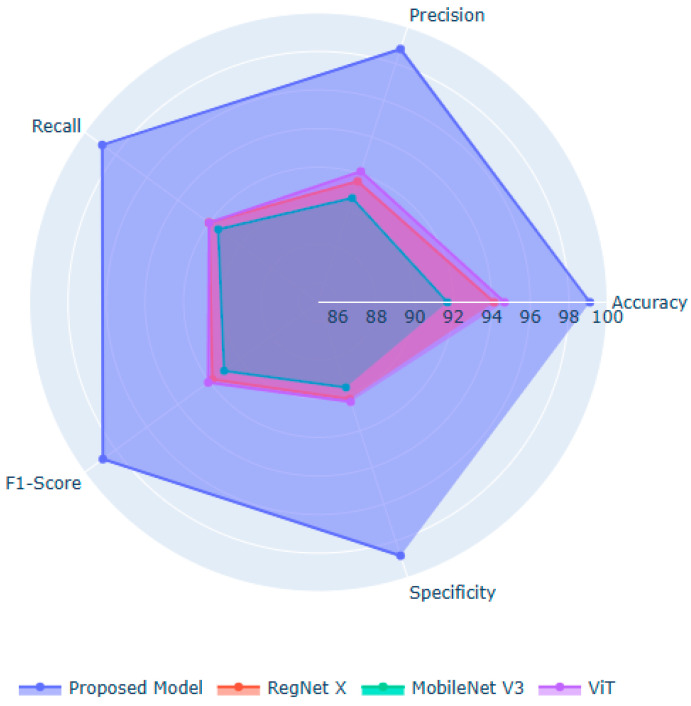
Findings of Performance Analysis (Proposed Model against Baseline Models).

Figure 5 illustrates the significant performance improvements achieved by the proposed model over the baseline models. RegNet X and MobileNet V3 are ideal for extracting localized spatial features. However, their performance is limited in modeling long-range dependencies and global facial structures, which are essential for classifying DS. ViT captures global context and relational cues between facial regions. Nonetheless, they lack the fine-grained local sensitivity needed for subtle phenotypic features. The potential of the proposed model lies in fusing the local detail extraction capabilities of the suggested feature extraction approaches. The introduction of the Linformer mechanism in the feature extraction process enables efficient modeling of long-range dependencies in the facial images with limited computational resources. Moreover, the BOHB-fine-tuned ExtraTrees deliver a robust decision boundary, enabling feature importance estimation through the SHAP values.

Table 5 demonstrates the superior classification performance of the proposed model through the ExtraTrees classifier, achieving the highest values across the key evaluation metrics. The proposed model outperforms Random Forest, XGBoost, Support Vector Machine, and a fully connected layer, underscoring the ability of the ExtraTrees classifier in handling the high-dimensional feature space. By introducing randomness in feature selection and split thresholds, the ExtraTrees classifier enhances the model’s diversity and reduces overfitting, ensuring clinical transparency and reliability.

Table 6 presents the significance and methodological advantages of the proposed model, demonstrating its robustness and reliability across diverse input samples. The moderate number of parameters and FLOPs indicates the effectiveness of the model’s design in maintaining a balance between representational power and computational efficiency. The proposed model is more efficient than RegNet X, ViT, and Linformer architectures, delivering remarkable results with low standard deviation and high confidence intervals. It maintains high prediction consistency with low loss across folds and samples. Compared to standalone CNNs and ViTs, it offers faster inference speed, supporting its applicability for large-scale studies and real-time clinical environment. MobileNet V3 requires minimal computational costs to classify the facial images. However, its limited feature representational power reduces the classification performance. The limited training time and inference speed demonstrate the importance of the proposed model in low-resource healthcare settings, serving as an effective DS screening tool. In addition, the incorporation of the adaptive attention mechanism and cross-demographic training addresses the limitations of RegNet X, MobileNet V3, and Linformer models, including limited global context understanding, the risk of underfitting in complex diagnoses, lower representational capacity, loss of expressiveness in attention compression, and weaker performance in isolation.

Karyotyping remains the gold standard for DS diagnosis, visualizing chromosomal abnormalities with near-perfect accuracy. However, it demands a specialized cytogenetic laboratory infrastructure and invasive sample collection. In addition, the delivery of findings may take several days. The related expense may be prohibitive for large-scale screening, especially in low- and middle-income countries. As a result, its application is typically restricted to confirmation diagnosis rather than universal early-life screening. In contrast, the proposed DS diagnosis is non-invasive, low-cost, and rapid, rendering it suitable for point-of-care and remote settings, requiring a standard digital camera or smartphone and limited computational resources. It produces near-instantaneous outputs without the need for specialized laboratory personnel. From a cost-effective perspective, efficient screening of large populations can be achieved through the proposed model, prioritizing high-risk cases for confirmatory karyotyping. The use of this personalized testing technique has the potential to lower the diagnostic cost per patient and maximize the utilization of laboratory resources.

Although the proposed model is not intended to replace genetic testing, its high sensitivity and specificity make it a useful triage tool in situations where karyotyping is technically possible but logistically constrained due to resource bottlenecks or geographical isolation. Through its role as an approachable first-line screening tool, the proposed model can complement karyotyping, allowing for earlier intervention program implementation.

**Figure 6:**
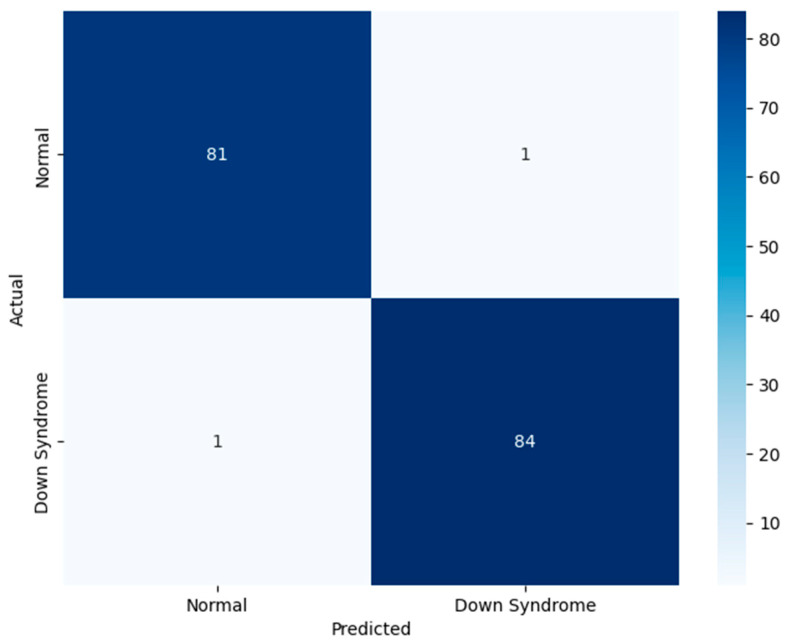
Confusion Matrix—DS Classification.

Figure 6 demonstrates the proposed model’s performance on the test set. The test set comprises unseen data, assessing the model’s real-time classification ability. The significance of the test set lies in its role as indicator of clinical readiness. The findings provide a transparent view of model’s behavior across normal and DS classes. The figure underscores the generalization capability of the model. The proposed model is highly accurate and free from bias toward either class, which is crucial for ethical deployment. Through the novel feature fusion strategy, the model captures local anatomical features and global facial symmetry patterns. The suggested adaptive attention-based fusion enhances the model’s diagnostic relevance, focusing on the crucial informative regions of the face during inference. The methodological emphasis on fairness and interpretability leads to the adoption of a responsible artificial intelligence-based application in pediatric healthcare settings.

**Figure 7:**
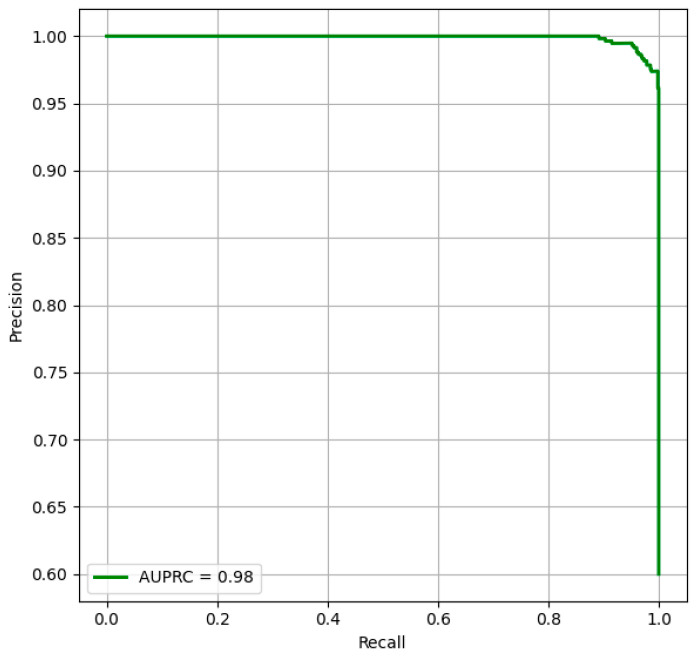
AUPRC—DS Classification.

Figure 7 provides the outcomes of the model’s performance by focusing on the relationship between precision and recall, reinforcing the model’s prediction power across threshold settings. The strong AUPRC performance stems from the innovative feature extraction and fusion strategies, enabling the model to identify fine-grained facial textures and global structural dependencies. Overall, the outcomes highlight the model’s potential as a trustworthy and generalizable tool for early DS diagnosis.

Table 7 provides transparent and clinically interpretable insights into the model’s decision-making process. The application of the SHAP values addresses the gap between the model’s predictions and human-understandable reasoning. For instance, the normal class predictions are associated with positive contributions of features, including chin length and increased eye distance. In contrast, features, such as reduced palpebral fissure length and nasal bridge heights, are contributing to the identification of the individuals with DS. In a binary classification setting, the lower confidence score for correctly predicted DS cases indicates the model’s strong prediction ability compared to the higher confidence score in predicting normal cases. The model’s reliability is reinforced by the confidence scores, rendering the proposed model actionable in real-world settings. The nuanced interpretability boosts clinical trust, supporting differential diagnosis in ambiguous cases.

Table 8 offers a comparative evaluation of the proposed DS classification against state-of-the-art approaches using the test set, demonstrating superior performance of the proposed DS screening model. By achieving an average accuracy of 99.10%, precision of 98.80%, recall of 98.87%, F1-score of 98.89%, and specificity of 98.81, the proposed model outperforms the existing approaches. The dependence on single-network architectures and traditional feature engineering approaches reduces the existing model’s performance in capturing fine-grained facial relationships. Although the recent models, including Islam and Shaikh [34] and Raza et al. [35], produced better outcomes, they lack generalizability. The absence of explainability or bias mitigation cause challenges in understanding their predictions or uncover potential shortcomings. In contrast, through the incorporation of different data, stratified cross-validation, fairness-aware optimization, and interpretable output, the proposed study overcomes these key shortcomings. The inherent limitations of VGG and CNN restrict their model performance in a real-time environment. The existing approaches based on end-to-end black box classifiers (ResNet-support vector machine, CNN-SVM, and VGG variants) lack model interpretability. The performance of the existing approaches is limited in capturing global spatial relationships, which are crucial for DS diagnosis. In addition, SVM models are unable to learn hierarchical features from raw facial images, restricting their capability to provide localized, visual, or class-wise interpretability. The proposed model significantly outperforms the existing approaches across classification performance, interpretability, and model design. The highest overall performance on a relatively large dataset highlights the importance of the proposed model.

## 5. Discussion

The study findings underscore the potential of proposed feature extraction, fusion, and classification techniques in detecting DS using OCT images. We have addressed the challenges with representational richness, model overfitting, and domain adaptability by focusing on improved feature fusion. Through the fusion of local and global descriptors, the proposed model enables clinicians to monitor the critical early stages of a child’s development. By shortening the timeline between early infancy and the formal diagnosis, families are provided with adequate opportunity to plan for treatments, educational programs, and medical interventions, mitigating long-term complications and enhancing the individuals’ quality of life. By incorporating a dual-branch feature extraction approach consisting of RegNetX–MobileNet V3 for localized structural learning and ViT–Linformer for global semantic representation, the proposed model improves the DS classification, enabling early DS identification. The model holistically interprets face morphology, identifying crucial DS features, including the shortening of the palpebral fissure, the flattening of the nasal bridge, and the presence of macroglossia. By dynamically weighing the multi-scale DS characteristics based on their diagnostic significance, the adaptive attention-based fusion module ensures that the model concentrates on medically essential characteristics rather than irrelevant variations. Unlike traditional gradient boosting and decision trees classifiers, the proposed fine-tuned ExtraTrees classifier classifies the features with optimal precision. To maintain a trade-off between model complexity and generalizability, BOHB allows for efficient selection of ExtraTrees’ hyperparameters, including tree depth, number of estimators, and feature subsets. In order to reduce overfitting and improve stability over a wide range of demographic groups, ExtraTrees involves the use of randomization in the feature selection and split thresholds processes, identifying the subtle morphological features associated with DS and normal individuals. Additionally, a robust interpretability layer is generated by combining the ExtraTrees classifier with SHAP values, highlighting the key features influencing the model’s decision. In a clinical setting, the model’s interpretability assists pediatricians and healthcare professionals in comprehending and trusting the decisions.

Optimal detection accuracy minimizes the need for expensive confirmatory testing in borderline instances, reducing the strain on healthcare infrastructures. With its lightweight architecture and use of a tree-based classifier, the proposed model is highly suitable for mobile Health deployments. As a means of facilitating early diagnosis and suggestion, it can be included in pediatric applications, clinical decision support systems, or telemedicine platforms. In addition to facilitating implementation in mobile applications, the interpretability aids in providing parents with early insights into clinically grounded decisions. Using facial phenotypes as a non-invasive diagnostic window, advancements in technology may be applied to enhance feature fusion for other developmental and congenital disorders. As a result, this study serves as a platform for future advancements in medical imaging and promotes rapid improvement in DS diagnosis.

Although the model demonstrates impressive performance in DS classification, several limitations should be acknowledged to ensure a balanced interpretation of its clinical utility. Dataset diversity and size remain key limitations. Subtle variations in craniofacial features may influence model generalizability. The extreme image variability in clinical or home settings may affect the model’s reliability. Capturing high-quality photographs of infants or children with mobility limitations is challenging, which in turn impacts early-stage use. The intermediate representations from RegNet X–MobileNet V3 and ViT-Linformer are challenging to interpret clinically, limiting trust among healthcare professionals unfamiliar with AI models, despite the transparency gains provided at the output level. The ExtraTrees classifier may introduce computational challenges when scaled to large datasets or embedded into real-time systems.

While this study focuses on DS detection, feature fusion applies to the broader domain of medical image analysis. Future research may focus on discovering additional face morphology-related chromosomal or genetic disorders using the proposed feature fusion approach. These improvements require further study in hardware compatibility and robust user interfaces to assist non-expert healthcare professionals. The combination of improved DS detection architecture, federated learning, privacy-preservation techniques, and increased interpretability tools enables DS screenings to be more accessible, precise, and user-friendly.

Non-invasive 3D ultrasound prenatal screening may reveal structural and phenotypic indicators of Down syndrome. However, operator competence, equipment availability, and image quality may restrict its practical value. Fetal position, maternal factors, and imaging capture variability might overlook subtle craniofacial or skeletal clues, resulting in uneven sensitivity across populations. In contrast, the proposed artificial intelligence-based facial image analysis approach is developed for postnatal detection. It makes use of high-resolution face morphology, which is more completely expressed after birth. This allows for highly discriminative feature extraction through the use of RegNet X and SWIN Transformer pipelines, rendering the model a useful and accessible resource for making confirmatory decisions on genetic testing and early intervention in the postnatal period.

The proposed approach provides the methodological groundwork for adapting to prenatal conditions by using feature extraction and attention-based fusion to 3D ultrasound data. Through this process, it has the potential to enhance the conventional interpretation of ultrasound by lowering the amount of observer variability and drawing attention to minor indicators that may not be apparent to clinicians, reducing the need for invasive treatments such as amniocentesis. Although this study targets postnatal detection, it addresses a critical gap in resource-limited environments and lays the groundwork for future prenatal screening applications.

When dealing with vulnerable groups like children and individuals with DS, facial image acquisition for AI-based diagnostics inherently involves sensitive ethical considerations. In this study, the facial images are obtained from publicly accessible datasets with open licenses with verified informed permission from participants or their legal guardians. In order to protect individuals’ privacy, we anonymized the images and deleted any identifiable information, such as file names, geotags, or embedded EXIF data, preventing misuse of biometric data and maintaining public trust in AI-based healthcare solutions. Although the present model focuses on binary categorization between DS and typical development, future versions may include subtype classification, discriminating between full trisomy 21, translocation, and mosaicism. Curated datasets annotated with karyotype-confirmed subtype labels and multi-branch neural network architectures are required to improve the proposed model’s classification ability, aiding clinical decision-making in tailored intervention planning.

Technical fairness safeguards, including SHAP-based interpretability and cross-demographic bias mitigation, are used in this research. However, these measures lack a comprehensive ethical framework. In order to improve the practical usability of the proposed DS diagnosis model, future research should investigate prenatal screening capabilities by expanding the model to evaluate three-dimensional ultrasound imaging, facilitating non-invasive and early-stage phenotypic testing. The use of multimodal data sources, such as genetic markers, motor skill assessments, and speech pattern analysis, has the potential to provide a holistic phenotyping approach, offering novel insights into the spectrum of DS presentations. Ethical considerations must continue to be a fundamental aspect of this technical evolution.

In order to maintain public trust and regulatory compliance, formulating clear guidelines is essential for the appropriate deployment of artificial intelligence in healthcare settings, encompassing privacy protection, secured data management, and transparent informed consent procedures. In addition, the fundamental design of the system may be customized to identify additional syndromes that have subtle or overlapping phenotypic characteristics, such as Williams’ syndrome or Noonan syndrome, increasing its utility in real-world pediatric care. By following these approaches, the proposed model can be an equitable, scalable, and trustworthy digital diagnostic assistance tool, supporting clinical processes in diverse healthcare settings.

The proposed model is intended to serve as a screening aid. It should be used ethically through physician-mediated decision support systems, with explicit parental agreement, and culturally appropriate counseling. In addition, robust governance mechanisms, stakeholder engagement, and continuous audits are essential to identify and address unforeseen implications in clinical settings.

## 6. Conclusions

In this study, a non-invasive DS screening model is developed using a hybrid feature extraction, adaptive attention-based fusion, and fine-tuned ExtraTrees classifier, achieving high-classification performance with limited computational resources. This study’s major contribution is the novel feature extraction approach using the hybrid CNNs-ViTs architecture, capturing the nuanced craniofacial DS features. The adaptive feature fusion and the use of fine-tuned ExtraTrees classifier enhance the model’s discriminative power. Another major contribution is the incorporation of explainable artificial intelligence approach, enabling the model’s interpretability. The proposed model addresses the limitations of existing DL models, enhancing diagnostic accuracy, ensuring clinical transparency, and facilitating demographic fairness, achieving an exceptional generalization accuracy of 99.10% and F1-score of 98.83% with limited computational resources. The integration of local feature extractors and global context learners identifies a rich spectrum of diagnostic indicators. Identifying key facial features, including palpebral fissure length, nasal bridge height, and chin length, associated with model’s prediction fosters clinical trust, particularly in clinical settings where expertise in genetics may be limited. The uncertainty analysis highlights the model’s reliability and robustness in classifying DS using the complex facial images, supporting the development of accessible, ethical, and scalable digital healthcare solutions. However, the study acknowledges certain limitations and future enhancements. Although the dataset provides sufficient images to train the model, the inclusion of underrepresented ethnic groups can improve the model’s generalizability and fairness. Exploring the incorporation of multimodal data, including speech, genetic data, speech samples, motor behavior, and clinical metadata with the proposed model may offer substantial phenotypic DS indicators. The reliance on two-dimensional photographic images may limit the model’s ability in capturing three-dimensional craniofacial features relevant for syndromic assessment. Furthermore, future work should focus on integrating attention visualization, enhancing the explainability of the fusion process. Prospective clinical studies are essential to evaluate the model’s capability in diverse healthcare settings. Exploring federated learning and privacy-preserving inference techniques can enable large-scale and secured training on globally distributed datasets.

## Figures and Tables

**Table 1:** Characteristics of the Categorized Dataset.

Age Group	Individuals with DS	Normal	Total Images
Infant (0–2 years)	424	406	830
Preschool (3–5 years)	310	315	625
School-age (6–10 years)	39	400	795
Adolescents (11–15)	375	374	749

**Table 2:** Model Implementation Setup.

Component	Specification
Operating System	Windows 11 Pro
RAM	32 GB
Framework	PyTorch, Torchvision, Scikit-Learn, and timm
Fusion Strategy	Adaptive attention-based feature fusion
Optimization	BOHB
Cross—Validation	Five—fold (stratified by class and ethnicity)
Augmentation	Applied on training folds only
Generalization	Conducted on the fifth fold

**Table 3:** Optimal Hyperparameter Settings.

Hyperparameter	Optimal Value
Learning rate	0.005
n_estimators	300
max_depth	20
min_samples_split	4
min_samples_leaf	2
Regularization	0.01
random_state	42 (fixed)

**Table 4:** Findings of Five-Fold Cross-Validation.

Folds	Accuracy	Precision	Recall	F1-Score	Specificity
1	99.32	98.89	98.58	98.73	99.17
2	98.84	99.24	99.37	99.30	98.45
3	98.36	98.47	99.19	98.83	97.98
4	99.24	98.96	98.77	98.86	98.15
5	99.10	98.80	98.87	98.83	98.81

**Table 5:** Findings of Performance Comparison (Proposed Model against Traditional Ensemble Models).

Model	Accuracy	Precision	Recall	F1-Score	Specificity
Proposed Model (ExtraTrees)	99.10	98.80	98.87	98.83	98.81
Random Forest	98.01	97.92	97.60	97.75	95.26
XGBoost	97.25	96.88	96.84	96.86	95.18
Support Vector Machine	96.29	96.50	96.11	96.30	96.27
Fully Connected Layer	96.42	96.93	96.88	96.90	95.90

**Table 6:** Findings of Uncertainty Analysis.

Models	Number of Parameters (in Millions)	Number of FLOPs	Standard Deviation	Confidence Interval	Loss	Training Time (CPU)	Inference Speed
Proposed Model	21.7	2.9 GFLOPs	0.0007	97.6–99.2	0.12	~2.8 h	0.24 s
RegNet X	29.5	2.9 GFLOPs	0.0017	94.7–96.9	0.29	~3.2 h	0.36 s
MobileNet V3	4.7	0.11 GFLOPs	0.0012	94.7–96.1	0.34	~2.1 h	0.21 s
ViT	81.2	15.2 GFLOPs	0.0021	96.1–98.4	0.22	~6.5 h	0.34 s

**Table 7:** Sample Inputs and Outputs with the SHAP Values.

Inputs	Ground Truth	Prediction	Confidence	SHAP Significance
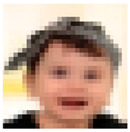	Normal	Normal	0.94	Chin length (+0.77) and Nasal width (+0.68)
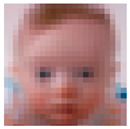	DS	DS	0.05	Palpebral fissure length (−0.81) and Nasal bridge height (−0.57)
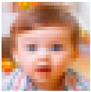	Normal	Normal	0.91	Eye distance (+0.74)
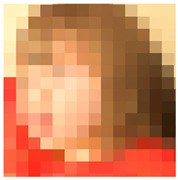	DS	DS	0.08	Mouth width (−0.53) and Midface height (−0.36)

**Table 8:** Findings of Comparative Analysis (Proposed Model against State-of-the-art Models).

Model	Accuracy (%)	Precision (%)	Recall (%)	F1-Score (%)	Specificity (%)
Proposed Model	99.10	98.80	98.87	98.83	98.81
Mittal et al. (2020) [28]	96.40	95.70	96.10	95.90	95.80
Pooch et al. (2020) [29]	96.90	96.10	95.80	95.95	96.20
Qin et al. (2020) [30]	97.30	96.80	96.00	96.40	97.10
Wang et al. (2022) [31]	97.50	97.00	97.10	97.00	97.10
Geremek and Szklanny (2021) [32]	96.70	95.90	96.50	96.20	95.90
Porras et al. (2021) [33]	97.10	96.30	96.10	96.20	96.70
Islam and Shaikh (2024) [34]	97.20	96.90	97.00	96.95	97.00
Raza et al. (2024) [35]	96.80	96.10	95.90	96.00	96.50

## Data Availability

The original contributions presented in the study are included in the article, further inquiries can be directed to the corresponding author.
